# The ICUPASS Study: Variability, Influencing Factors, and Clinician Stress in ICU Admission Decisions

**DOI:** 10.7759/cureus.111068

**Published:** 2026-06-18

**Authors:** Cyril Chacko, Girish Kanna, Shaveta Devasar, Amit Sharma, Thomas Fisher, Jagatar S Pooni, Andrew Macduff, Shameer Gopal, Kesaven Dhamodaran, Suresh Renukappa, Suresh Subashini, Suneesh Thilak, Krishna Navaneetham, Syed Haque, Palliniswamy Matheswaran, Nasa Prashant, Tonny Veenith

**Affiliations:** 1 Critical Care Medicine, Royal Wolverhampton Hospital, Wolverhampton, GBR; 2 Critical Care Medicine, The Royal Wolverhampton NHS Trust, Wolverhampton, GBR; 3 Anesthesia and Critical Care, Royal Stoke University Hospital, London, GBR; 4 Critical Care, The Royal Wolverhampton NHS Trust, Wolverhampton, GBR; 5 Faculty of Science and Engineering, University of Wolverhampton, Wolverhampton, GBR

**Keywords:** clinical decision-making, clinical variability, cross sectional studies, futility, health care disparities, intensive care unit, practice variation, psychological stress, triage, vignette based surveys and questionnaires

## Abstract

Objectives

The objectives of this study were to quantify the variability in intensive care unit (ICU) admission decisions, identify influencing factors, and measure the associated stress among different groups of healthcare professionals.

Design

This was a cross-sectional survey using 10 standardised clinical vignettes.

Setting

The study was conducted in ICUs in the West Midlands, UK.

Participants

The study included 84 ICU professionals, categorised as decision-makers ([DMs] consultants and senior registrars, n=47) or non-decision-makers ([NDMs] other clinical staff, n=37).

Main outcome measures

For each vignette, respondents rated the likelihood of admission, associated stress, and likelihood of admission with unlimited resources on a 10-point Likert scale. The primary reason for non-admission was also selected.

Results

Admission likelihood was heterogeneous across all vignettes (median 5, IQR 6), with no significant difference between DMs and NDMs. DMs reported significantly lower stress than NDMs (median 4 vs 5, p<0.001). Clinically ambiguous vignettes showed the highest variability and stress. Counterintuitively, admission likelihood was significantly lower under a hypothetical unlimited-resources scenario (median 3 vs 5, p<0.001). "Futility" was the most common reason cited for non-admission.

Conclusions

Substantial variability and stress are inherent to ICU admission decisions, even among experienced clinicians. Subjective clinician factors shape decisions alongside objective patient data, underscoring the need for decision-support tools in ambiguous cases and strategies to ease the psychological burden on clinical staff.

## Introduction

Intensive care units (ICUs) represent a critical and scarce healthcare resource, providing advanced organ support and monitoring for the most severely ill patients. The decision to admit a patient to the ICU is crucial in a patient's clinical trajectory, often determining the scope of care and influencing outcomes [1-3]. As such, intensivists and other critical care professionals act as essential gatekeepers to this high-cost, high-impact environment [4].

The complexity of this role has increased substantially. The patient population considered for ICU admission is progressively older and characterised by a greater burden of chronic comorbidities, multi-morbidity, and frailty [5,6]. This clinical complexity introduces significant prognostic uncertainty, challenging clinicians' ability to accurately predict which patients will derive meaningful benefit from intensive care. This inherent uncertainty is a primary driver of variability in practice, as clinicians with similar training may reach different admission decisions for the same patient [7-9].

This variability is a well-documented phenomenon influenced by system-level factors, such as bed capacity, local admission policies, and seasonal surge pressures, which can alter the admission threshold [10,11]. Patient-specific factors, including clinical severity, age, and pre-existing comorbidities, are central to the decision [12]. However, clinician-specific factors, such as experience, speciality, risk tolerance, and cognitive biases, also play a significant, though less quantifiable, role [12,13].

While practice variability is a known challenge, the psychological impact on the decision-maker (DM) remains a less explored dimension of ICU care. The constant pressure of making high-stakes decisions under uncertainty contributes to cognitive overload and emotional exhaustion, which, in turn, contribute to burnout, which is prevalent among ICU professionals [14,15]. The stress associated with borderline admissions, where the potential for benefit is equivocal, places a particular burden on clinicians [16]. This study was designed to address this gap by systematically investigating the factors that contribute to variability in ICU admission decisions and, for the first time, quantifying the stress associated with this fundamental clinical task.

The primary aim of this study was to quantify the variability in ICU admission decisions among a multi-professional cohort of ICU clinicians using standardised clinical vignettes. Secondary aims were to compare decision-making patterns and stress levels between designated DMs and other clinical staff, to identify the primary reasons cited for declining ICU admission, and to explore the relationship between the likelihood of making a decision, clinician stress, and hypothetical resource availability. We hypothesised that significant variability would exist across all respondent groups, that designated DMs would report lower stress levels than non-decision-makers (NDMs), and that cases pre-defined as clinically ambiguous would elicit the highest levels of variability and stress.

## Materials and methods

Study design and survey development

A cross-sectional, vignette-based survey was conducted [17,18] among healthcare professionals working in ICUs at National Health Service (NHS) trusts in the West Midlands region of the United Kingdom between October and December 2025. The survey instrument consisted of 10 hypothetical clinical vignettes, each describing a distinct and realistic acute care scenario that would typically prompt a referral for an ICU admission decision. The vignettes were composed by the authors based on their clinical experience. The full set of vignettes is provided in Appendix A. This cross-sectional study is reported in accordance with the Strengthening the Reporting of Observational Studies in Epidemiology (STROBE) statement [19].

A key feature of the study design was the a priori categorisation of vignettes. Before survey distribution, a panel of eight experienced ICU consultants, who were not participants in the main study, independently reviewed each vignette and classified it into one of three groups based on the anticipated consensus for admission. Group assignment was determined by majority agreement, with any discordant classifications resolved through discussion. Vignettes that achieved full consensus without discussion were classified directly, whereas those requiring deliberation were noted to reflect genuine clinical ambiguity.

The a priori vignette categories were as follows:

1. Group A (clear admit): four vignettes where ICU admission was deemed clearly indicated (cases 1, 3, 4, 8)

2. Group B (clear reject): three vignettes where admission was deemed clearly not indicated or potentially harmful (cases 2, 5, 10)

3. Group C (ambiguous): three vignettes designed to be borderline or contentious, where the decision could reasonably go either way (cases 6, 7, 9)

Data collection and variables

For each of the 10 vignettes, the survey captured responses to four key questions. Respondents used a 10-point Likert scale (where 1 indicated very low likelihood/stress and 10 indicated very high likelihood/stress) to rate the following. The 10-point Likert scale was chosen over other smaller scales to capture finer gradations in admission likelihood and stress, particularly for vignettes anticipated to generate heterogeneous responses, consistent with prior applications in clinical decision-making research [20-22]:

1. Likelihood of admission: the likelihood that the respondent would admit the patient to their ICU under current typical conditions

2. .Decisional stress: the level of personal stress the respondent associated with making that specific admission decision

3. Likelihood with unlimited resources: the likelihood that the respondent would admit the patient in a hypothetical scenario with no constraints on bed availability or staffing

4. Primary reason for non-admission: a single multiple-choice question capturing the primary perceived reason for non-admission, with options comprising the following: futility, high six-month mortality, multi-morbidity, patient too well, none, and others. Respondents selected one reason per vignette

The survey also collected demographic data on respondents' professional role and primary speciality (intensive care medicine [ICM] or non-ICM).

 Recruitment and sampling

A census sampling approach was used to target all eligible clinical staff in participating ICUs during the study period. Participating units were Sandwell and West Birmingham Hospitals NHS Trust, The Dudley Group NHS Foundation Trust, The Royal Wolverhampton NHS Trust, Walsall Healthcare NHS Trust, and University Hospitals of Birmingham NHS Trust. Responses from outside these regions were excluded from analysis.

The questionnaire was sent to each participating unit through trainee leads in ICM, and all eligible individuals were invited to participate. The survey was distributed electronically via a secure online platform (Google Forms) with a unique access link circulated through departmental email lists. Participation was entirely voluntary and anonymous; no personally identifiable information was collected. They were only asked to provide an email if they wanted feedback about the results. A single reminder was sent two weeks after the initial distribution. The survey was closed after four weeks.

Sample size

No formal a priori sample size calculation was performed, as this study employed a census design in which all eligible ICU professionals within the participating units were invited. This approach is consistent with established methodology for vignette-based survey studies conducted within a defined institutional population, where the objective is to capture the full range of perspectives within a bounded clinical community rather than to achieve a predetermined statistical power for a primary comparative endpoint [23]. The final analytic sample of 83 participants generated 830 complete vignette-level responses across 10 scenarios. This volume of observations was considered sufficient to characterise the distribution of Likert-scale responses within and between subgroups, to detect a statistically significant difference in stress scores between DMs and NDMs (Mann-Whitney U, p<0.001), and to apply Bonferroni-corrected comparisons across 10 cases. The sample size and its limitations regarding subgroup analyses and generalisability are acknowledged in the Discussion section.

Participant cohort

Inclusion criteria were current employment as a doctor, nurse, or advanced critical care practitioner (ACCP) within an ICU at a participating NHS Trust, and active clinical involvement in ICU care at the time of survey distribution. There were no exclusion criteria beyond non-clinical or administrative roles. Respondents with incomplete responses (defined as failure to complete the majority of vignette questions) were excluded from analysis; one respondent from the NDM group was excluded on this basis, yielding a final analytic sample of 83 participants. Respondents were categorised into two primary groups reflecting their typical level of responsibility for the final admission decision:

1. DMs: ICU consultants and senior ICU residents (postgraduate training year 4 and above)

2. NDMs: all other respondents, such as junior doctors, ICU nurses, and ACCPs

Statistical analysis

Statistical analysis was conducted using Jamovi (Version 2.6). Due to the ordinal nature and non-normal distribution of Likert-scale data, descriptive statistics are presented as medians and interquartile ranges (IQRs). Group comparisons between DMs and NDMs were performed using the Mann-Whitney U test, with U statistics reported alongside p-values. The relationship between ordinal variables, such as admission likelihood and stress, was assessed using Spearman's rank correlation coefficient (rho). A Friedman test was used for repeated-measures analysis of the primary variables. Chi-square tests were used to compare the proportions of categorical reasons for non-admission between groups, with chi-square (chi2) statistics and degrees of freedom (df) reported. For the case-by-case analysis involving 10 comparisons, a Bonferroni correction was applied, with statistical significance set at p<0.005. Incomplete responses were excluded from the relevant vignette-level analyses.

## Results

Cohort characteristics

A total of 84 healthcare professionals were invited to participate, of whom one NDM was excluded owing to missing responses across the majority of vignettes. The final cohort generated 830 complete vignette-level responses. Of these, 47 (56.6%) were classified as DMs, all of whom were senior ICU doctors. The remaining 36 (43.4%) were classified as NDMs, comprising ICU nurses (n=17, 47.2% of NDMs), ICU residents (n=8, 22.2% of NDMs), non-ICU doctors (n=7, 19.4% of NDMs), and ACCPs (n=4, 11.1% of NDMs). Full demographic data are presented in Table 1.

**Table 1:** Participant demographics and professional roles. ACCP, advanced critical care practitioner; DM, decision-makers; ICU, intensive care unit; NDM, non-decision makers

Role	Counts	% of Total
ICU doctor senior (DM)	47	56.6%
ICU nurse (NDM)	17	20.5%
ICU resident (NDM)	8	9.6%
Non-ICU doctor (NDM)	7	8.4%
ACCP (NDM)	4	4.8%
Total	83	100.0%

Heterogeneity in admission likelihood by vignette category

Analysis by the a priori vignette categories revealed patterns in admission likelihood broadly consistent with the expert consensus panel's classifications, with variability differing by group and case type.

Decision-makers

Among DMs, group A (clear admit) cases yielded a median admission likelihood of 7 (IQR: 4). Group B (clear reject) cases had a lower median of 2 (IQR: 2). Group C (ambiguous) cases showed the greatest spread, with a median of 4 (IQR: 4).

Non-decision-makers

NDMs showed comparable median values to DMs for group A cases (median 7, IQR: 4). For group B, the median was 3 (IQR: 6). Group C responses had a median of 5 (IQR: 5).

Decisional stress

Making ICU admission decisions was associated with stress across all participants and vignettes, with an overall median stress score of 5 (IQR: 4). DMs reported a lower overall median stress score than NDMs (4 [IQR: 4] vs 5 [IQR: 4]; Mann-Whitney U=5,103, p<0.001). By vignette category, DM stress was highest for group A (median 5, IQR: 4) and group C (median 5, IQR: 4) cases, and lowest for group B (median 2, IQR: 4). NDM stress scores were consistently higher across all categories: group A median 6 (IQR: 4), group B median 4 (IQR: 5), and group C median 6 (IQR: 4).

Respondents whose primary speciality was ICM reported lower stress than those from non-ICM backgrounds (median 4 vs 5; Mann-Whitney U, p<0.001). A weak but statistically significant positive correlation was observed between admission likelihood and stress (Spearman's rho=0.207, p<0.001); the direction of this association cannot be determined from this cross-sectional design.

Analysis by a priori vignette category

Findings by vignette category are summarised in Table 2. Group C (ambiguous) cases were associated with the widest IQRs for likelihood and the highest median stress scores for both DMs and NDMs. Group B (clear reject) cases showed the lowest admission likelihood and the lowest stress scores across both groups.

**Table 2:** Analysis of responses by a priori vignette category. Data presented as median (IQR). Group A (clear admit): cases 1, 3, 4, 8. Group B (clear reject): cases 2, 5, 10. Group C (ambiguous): cases 6, 7, 9. Likelihood: rated probability of ICU admission (0-10 NRS). Stress: self-reported decisional stress (0-10 NRS). Unlimited resources: rated likelihood of admission assuming no resource constraints (0-10 NRS). The p-values and U statistics were derived from the Mann-Whitney U test comparing DMs versus NDMs within each category. DM, decision-maker; ICU, intensive care unit; IQR, interquartile range; NDM, non-decision-maker; NRS, numerical rating scale

	Group A: Clear Admit	Group B: Clear Reject	Group C: Ambiguous	Overall	DM vs NDM (p; U)
Likelihood					
DMs	7 (4)	2 (2)	4 (4)	5 (5)	p<0.001; U=4,821
NDMs	7 (4)	3 (6)	5 (5)	5 (6)	—
Stress					
DMs	5 (4)	2 (4)	5 (4)	4 (4)	p<0.001; U=5,103
NDMs	6 (4)	4 (5)	6 (4)	5 (4)	—
Unlimited resources					
DMs	7 (6)	2 (3)	3 (6)	4 (7)	p=0.031; U=5,892
NDMs	6 (6)	3 (7)	4 (7)	3 (7)	—

Influence of resource constraints

The overall likelihood of admission was lower under hypothetical unlimited resource conditions than under current conditions (median 3 [IQR: 7] vs 5 [IQR: 6]; Wilcoxon signed-rank, p<0.001). This pattern was consistent across both DMs and NDMs and across all three vignette categories (Table 2).

Case-by-case analysis of admission likelihood

Case-level comparisons between DMs and NDMs are presented in Table 3. After Bonferroni correction (threshold p<0.005), one vignette demonstrated a statistically significant between-group difference: case 2, a group B vignette, in which DMs assigned a lower admission likelihood than NDMs (median 1 vs 3; Mann-Whitney U=554, p=0.002). A further nominally significant difference was observed for case 8 (p=0.031), which did not survive Bonferroni correction.

**Table 3:** Likelihood of ICU admission by case and respondent group. Data presented as median (IQR). Likelihood scored on a 0-10 NRS (0 = Definitely would not admit; 10 = Definitely would admit). Admit: pre-classified as favouring ICU admission. Reject: pre-classified as favouring non-admission. Ambiguous: clinical equipoise. p-values and U statistics were derived from the Mann-Whitney U test. Bonferroni-corrected threshold: p<0.005. *Statistically significant after Bonferroni correction. DM, decision-maker; ICU, intensive care unit; IQR, interquartile range; NDM, non-decision-maker

Case	Category	DMs Median (IQR)	NDMs Median (IQR)	p-Value	Test Statistic
1	Admit	6 (5)	7 (5)	0.553	U=765
2	Reject	1 (1)	3 (7)	0.002*	U=554
3	Admit	7 (6)	6 (6)	0.621	U=822
4	Admit	8 (2.5)	8 (5)	0.953	U=849
5	Reject	2 (3)	4 (7)	0.204	U=714
6	Ambiguous	2 (4.5)	3 (5)	0.889	U=843
7	Ambiguous	5 (5.5)	7 (6)	0.757	U=831
8	Admit	8 (4.5)	5 (5)	0.031	U=601
9	Ambiguous	4 (4)	4 (3)	0.689	U=815
10	Reject	2 (2)	3 (6)	0.206	U=711

Reasons for non-admission

Across all 10 vignettes and 83 respondents (830 vignette-level responses: 470 from DMs [47 x 10] and 360 from NDMs [36 x 10]), the most frequently cited reason for non-admission was futility (269/830, 32.4%), followed by multi-morbidity (163/830, 19.6%) and high six-month mortality (120/830, 14.5%). The distribution of stated reasons did not differ significantly between DMs and NDMs (chi2=4.12, df=5, p=0.53). Frequency data by group are presented in Table 4.

**Table 4:** Frequency distribution of stated reasons for non-admission by respondent group. Note: *n* denotes the total number of responses in the groups (not unique respondents), reflecting all vignette-level answers submitted within each group. Futility: clinician judged that ICU admission would not alter the patient's outcome. High six-month mortality: anticipated mortality at six months deemed too high to justify admission. Multi-morbidity: burden of comorbid conditions considered a contraindication to ICU-level care. Too well: patient's acuity deemed insufficient to warrant ICU admission. None: no specific reason selected; Others: reasons not captured by predefined categories. Decision-makers are clinicians with primary responsibility for ICU admission decisions. Non-decision-makers are all other respondents. ICU, intensive care unit

Reason	Decision-Makers (n=470)	Non-Decision-Makers (n=360)	Total (n=830)
Futility	148	121	269
High six-month mortality	76	44	120
Multi-morbidity	87	76	163
None	66	54	120
Others	36	23	59
Too well	57	42	99

## Discussion

This study provides a quantitative assessment of the variability, stress, and reasoning associated with ICU admission decisions in a multi-professional cohort. The findings confirm that the process of ICU gatekeeping is characterised by heterogeneity and is a source of stress for clinicians [24].

The most compelling finding is the scale of variability in admission likelihood, which persisted even among the most experienced cohort of senior ICU doctors. An IQR of 6 on a 10-point scale indicates a lack of consensus, suggesting that for many clinical scenarios, there is no single, universally accepted correct decision. This challenges the notion that clinical expertise necessarily leads to uniformity. Instead, it appears that experience equips clinicians with a set of individual heuristics and a personal calibration of risk versus benefit [25-28]. This implies that a patient's access to critical care may be substantially influenced by the on-call clinician's individual philosophy and risk tolerance, raising important questions about the equity of access to critical care.

A second key finding is the quantifiable stress associated with these decisions, as well as the transparent gradient that exists across the clinical team. The observation that NDMs, including nurses, junior doctors, and ACCPs, experience higher stress than their senior DM colleagues is noteworthy. This is unlikely to reflect the stress of ultimate responsibility; rather, it may represent the moral distress that can arise from disagreeing with a decision, feeling that a patient is receiving inappropriate care (either too much or too little), or lacking the agency to influence the outcome. This stress differential represents a potential source of inter-professional friction and may be a hidden contributor to burnout and staff turnover within the wider ICU team. While DMs reported lower stress, a median score of 4 is not trivial and underscores that even for seasoned practitioners, these decisions carry a persistent emotional and cognitive weight.

The analysis by vignette category further refines this understanding. As hypothesised, the clinically ambiguous cases were the epicentre of both disagreement (highest variability) and distress (highest stress scores). This is logical, as decisions at the margins of benefit are the most cognitively and ethically demanding [29]. This finding has direct practical implications, suggesting that efforts to improve decision-making, such as the development of clinical pathways or decision-support tools, should be targeted specifically at this grey zone of borderline admissions rather than at cases where consensus is already high [29].

Perhaps the most thought-provoking result is the resource paradox: the finding that clinicians would be less likely to admit patients under hypothetical conditions of unlimited resources. The conventional assumption is that resource scarcity is a primary barrier to admission. This result suggests a more complex reality. It may be that, in the current system, the ICU is often used as a safety net for deteriorating patients who could be managed in a lower-acuity setting if it were adequately staffed and equipped [30]. In a hypothetical world of unlimited resources, clinicians may envision an ideal system with robust high-dependency units or enhanced ward-based care, making ICU admission unnecessary for a subset of patients currently admitted because safer alternatives are lacking. This reframes some ICU admissions not as a definitive clinical need but as a response to system-wide capacity deficits [30].

Finally, the frequent citation of futility as a reason for non-admission highlights a critical challenge in modern critical care. Futility is an inherently subjective, value-laden concept [31]. The fact that clinicians cited futility in cases where there was still wide disagreement on admission likelihood demonstrates that there is no shared operational definition of the term. One clinician's futile intervention is another's worthwhile trial of therapy [31]. This underscores the need for improved communication frameworks and a greater emphasis on shared decision-making with patients and families to establish clear goals of care, which should precede any unilateral declaration of futility [32].

The challenges of information overload and cognitive burden are central to the stress and variability observed in this study. The modern ICU generates vast amounts of complex, often conflicting data that clinicians must rapidly synthesise to make optimal care decisions. Emerging technologies, particularly artificial intelligence (AI) and large language models (LLMs), present a potential pathway to mitigate these challenges. LLMs have demonstrated efficacy at processing and summarising large volumes of unstructured data, such as clinical notes and laboratory results, into concise, relevant reports. This capability for cognitive offloading can streamline information processing and reduce clinicians' cognitive load [33], allowing clinicians to focus more on direct patient care and complex decision-making. By providing evidence-based decision support and automating administrative tasks, these AI tools could help standardise care in ambiguous cases, alleviate some of the psychological burden, and potentially reduce burnout rates associated with high-stakes clinical judgements.

Strengths and limitations

This study has several strengths, including the use of standardised clinical vignettes to eliminate patient-specific confounders. The inclusion of a multi-professional cohort and the a priori expert categorisation of vignettes allowed for a nuanced analysis. However, some limitations must be acknowledged. The study was conducted in a single UK geographical region; practice patterns may differ in other regions or healthcare systems, limiting the generalisability of these findings. The use of hypothetical vignettes, while necessary for standardisation, cannot fully replicate the emotional weight and complex human factors, including fatigue, interpersonal dynamics, and time pressure, of real-time clinical decision-making. Lastly, the vignettes did not include information about patient and family wishes, which are a crucial component of real-world admission discussions.

## Conclusions

The decision to admit a patient to the ICU is characterised by profound inter-clinician variability and is a significant source of stress for the entire clinical team. This study demonstrates that these critical decisions are influenced not only by objective clinical data but also by subjective clinician factors, individual interpretations of concepts such as futility, and systemic resource pressures. The high degree of variability, even among experts, and the associated psychological burden on staff underscore an urgent need to develop decision-support tools for ambiguous cases, strengthen inter-professional communication strategies, and further investigate the complex interplay between clinical judgement, ethics, and resource allocation in the modern ICU.

## Appendices

Appendix A

Subject: Request to complete the Intensive Care Unit Patient Acceptance Survey and Associated Stress (ICUPASS) study.

Dear Colleagues,

I hope this email finds you well. As professionals working in the challenging environment of intensive care units (ICUs), we understand the immense responsibilities and stress associated with caring for our critically ill patients. Our commitment to patient care is unwavering, but it is essential that we also address the impact of our work on our well-being.

Recent research has highlighted the prevalence of burnout among healthcare providers, particularly those working in critical care settings. Burnout syndrome, characterised by emotional exhaustion, depersonalisation, and reduced personal accomplishment, can significantly affect both clinicians and patients. The ICU, with its unique demands and high patient mortality, is particularly susceptible to burnout. The increased incidence of decision-making fatigue is instrumental in the increased incidence of burnout. We have initiated a survey to understand better the variability in ICU acceptance practices and its association with stress, which results in burnout. By participating, you will contribute valuable insights that can inform strategies to mitigate burnout and enhance fulfilment among ICU clinicians.

This survey has 10 hypothetical patients, which you are asked to review for admission to ICU. We want your opinion on whether you will admit this patient to the ICU when this patient is competing for our last bed left in the ICU; we are collecting data on the probability of stress and admission on a Likert scale. We will repeat this survey at least three times to find the intersubject and within-subject variability of the decisions and the stress associated with each decision.

Why Your Participation Matters: Your responses will help identify patterns and best practices related to ICU acceptance. This knowledge will guide evidence-based interventions to improve our work environment. By understanding the factors contributing to stress and burnout, we can implement targeted interventions such as tightening the guidelines to reduce their impact on our well-being. The more clinicians participate, the stronger our collective voice becomes. Together, we can drive positive change within our profession.

How to Participate: Please take a few minutes to complete the survey using the link provided below. Your responses will remain confidential, and aggregated data will only be used for research purposes.

Survey Link here

Deadline: We kindly request your participation by [XXXXXXXX].

Your input is invaluable, and we appreciate your commitment to advancing our field.

I appreciate your dedication to patient care and willingness to contribute to this critical research. Let us work together to create a healthier and more supportive ICU environment.

Sincerely,

Consultants to sign their names here

1. An 85-year-old man is referred from the resuscitation room with drowsiness. The patient was recently discharged from the hospital after being treated for bilateral pneumonia. He has a past medical history of type 2 diabetes, COPD, and hypertension. His wife feels that he has not been very compliant with his treatment. On assessment: he is clinically dry and has a prolonged CRT (4 secs), HR 120, BP 80/65 mmHg, SpO_2_ 92% on room air, GCS 11/15, temp 34.1, urine output <0.5 mL/kg/hr. A venous blood gas shows pH 7.20, glucose 46 mmol/L, and blood ketones are recorded as 0.1 mmol/L.  Other investigations: S. osmolality >350 mOsm, sodium >162 mmol/L. CT head normal.

2. A 27-year-old is referred from the resuscitation room with drowsiness. He has a past medical history of type 1 diabetes mellitus. On examination, his respiratory rate is 24 breaths per minute, SpO_2_ is 94% on room air with normal breath sounds; he has cool peripheries and a GCS of 11. A venous blood gas is taken; this reveals pH 7.27, PCO_2_ 3.0 kPa, HCO_3_ 14 mmol/L, and lactate 1.7 mmol/L. Blood glucose is 27.9 mmol/L. Blood ketones is 4mmol/L.

3. A 39-year-old is referred from the resuscitation room with weight loss, fever, night sweats, and rapidly progressive abdominal swelling for three months. ABG shows severe metabolic acidosis (pH 6.9, base excess of -17) of unknown aetiology. She has extensive lymphadenopathy, with a possible diagnosis of disseminated TB or Lymphoma. She has a history (2 years back) of macrophage activation syndrome after being treated for rheumatoid arthritis. She has a good functional status and works in a nursing home. On examination, her respiratory rate is 34 breaths per minute, SpO_2_ 94% on room air, breath sounds are vesicular with no added sounds; she has cool peripheries and GCS of 11 (E2V4M5).

4. A 33-year-old woman is referred to ICU for hyperlactataemia. She has known sickle cell disease and a history of recurrent DVT, pulmonary hypertension, and spontaneous intracranial haemorrhage. She lives alone, and her family help with cleaning and shopping. She can walk about 15 yards before she is breathless and has to stop. When she was admitted to your hospital a year ago, she had been refused admission to the ICU for recurrent seizures due to a poor pre-morbid functional state. She has been referred as she is hypoxic, with saturations of 60% on room air with lactic acidosis (7 mmol/L). Her BP is 110/60. She is confused but cooperative. Her CXR shows bilateral infiltrates.

5. An 81-year-old man is referred from the resuscitation room. He was admitted with a headache and right-sided weakness. His GCS was 6 (E1, M3, V2). He was intubated in resus by the ED team. A CT scan showed an extensive left intracerebral haemorrhage with mass effect and early tonsillar herniation. He has a history of IHD and AF. He takes warfarin, bisoprolol, ramipril, and atorvastatin. He lives with his wife and is usually independent of ADLs. He has been referred to the neurosurgical team, who say he is for conservative management.

6. An 85-year-old man referred from the ward for consideration of non-invasive ventilation who has diabetes, hypertension, pulmonary embolism, and osteoarthritis was admitted to the hospital with abdominal pain. Due to his osteoarthritis, his ability to move is limited. He had a laparotomy and appendectomy but experienced complications of ileus and an intrabdominal collection. As a result, he has been in the hospital for over two weeks with minimal improvement in his condition. He currently has a Respect form in place. He has been referred to the ICU for type 1 respiratory failure caused by hospital-acquired pneumonia and pleural effusion.

7. A 49-year-old man was referred from resus with indwelling urinary catheter bleeding and severe hyponatraemia (Na+ 104 mmol/L) with three seizures. He has previous admissions with hyponatraemia, alcohol excess, permanent catheter in situ, bed bound due to likely a motor neuron disease.

8. A 66-year-old man was referred from resus with chest pain and agitation. He has a past medical history of type 2 diabetes mellitus, hypertension, ischaemic heart disease (with coronary stents), and an implantable cardiac electronic device (CRT-D). He is ordinarily active, continues working as a cook, and has been well until two days ago. His regular medications are aspirin, bisoprolol, clopidogrel, dapagliflozin, metformin, rosuvastatin, and sacubitril with valsartan. On examination, his respiratory rate is 34 breaths per minute with a sats of 91% on room air, his heart rate is 118 beats per minute, and his blood pressure is 84/52 mmHg. He has cool peripheries and a central capillary refill time of 4 seconds. He is intermittently agitated with a GCS of 11 (E2, V4, M5), blood gas pH <6.80, PaO_2_ 11.1 kPa, PaCO_2_ 2.3 kPa, Na+ 134 mmol/L, K+ 4.1 mmol/L, Cl- 90 mmol/L, glucose 8.3 mmol/L, lactate 15.4 mmol/L, BE ‘incalculable’, HCO_3_- ‘incalculable’, capillary ketones read >6 mmol/L.  An ECG shows sinus tachycardia. A bedside echocardiogram shows severe generalised biventricular systolic dysfunction.

9. A 37-year-old male patient is referred to ICU with a diagnosis of lymphoma on chemotherapy. He has developed severe electrolyte abnormalities following his chemo. His calcium is 0.6 mmol/L and magnesium is 0.4 mmol/L. Trust protocol says he needs to have a monitored bed for his calcium and magnesium correction to monitor for arrhythmia; none is available in the hospital apart from the ICU, and the treating doctor wants him to come to the ICU for electrolyte correction.

10. A 21-year-old woman has been on follow-up with various neurologists for functional epilepsy for the past two years. She also has autism spectrum disorder. She has had MRI, LP, and EEG, all within normal limits. ICU reg is called to the ward to assess the patient, who is found to have seizures and has dropped her GCS for the past 6-7 hours. Her HR is 90 and BP is 120/80. She has no movements following a trapezius squeeze. She goes to have a CT scan that shows no abnormality. She is maintaining her airway and is saturating 97% on room air. The family is angry at the ICU and ward teams and wants this patient to go to the ICU.
